# Association Between Serum Fluoride and Urine Fluoride Levels and Hemoglobin Levels in Pregnant Women: A Cross-Sectional Study in Rural Telangana, India

**DOI:** 10.7759/cureus.103839

**Published:** 2026-02-18

**Authors:** Mudavath Nikhil, Priyanka Das, Gunvanti Rathod, Sangeetha Sampath, Aparna V Bhongir

**Affiliations:** 1 Biochemistry, All India Institute of Medical Sciences, Bibinagar, Bibinagar, IND; 2 Pathology and Laboratory Medicine, All India Institute of Medical Sciences, Bibinagar, Bibinagar, IND

**Keywords:** anemia, hemoglobin, pregnant women, serum fluoride, urine fluoride, urine/serum fluoride ratio

## Abstract

Background

Anemia affects a substantial proportion of the global population, with pregnant women being particularly vulnerable. In regions with high fluoride levels in drinking water, fluoride exposure is known to impair nutrient absorption, potentially exacerbating anemia.

Objective

This study aimed to assess the correlation between serum and urinary fluoride levels and hemoglobin concentration in pregnant women, while controlling for other known causes of anemia.

Methods

A cross-sectional observational study was conducted on 98 pregnant women (gestational age <12 weeks) from high-fluoride villages (fluoride >4 ppm) and low-fluoride villages (fluoride ≤1.5 ppm) in the Yadadri Bhuvanagiri District of Telangana, India. Serum and urine fluoride levels were measured using an ion-selective electrode, and hemoglobin was analyzed using a three-part hematology analyzer. Statistical analyses included Pearson’s correlation, multivariate linear regression, and principal component analysis.

Results

Mean hemoglobin levels were slightly lower in the high-fluoride group (HFG: 11.80 ± 1.46 g/dL) than in the low-fluoride group (LFG: 11.98 ± 1.46 g/dL), with a higher prevalence of anemia in the HFG (24.48%) compared to the LFG (14.28%), though these differences were not statistically significant. Urine fluoride levels were higher in the HFG (1.28 ± 0.81 ppm) than in the LFG (1.09 ± 0.89 ppm; p > 0.05), while serum fluoride levels were marginally lower in the HFG (0.37 ± 0.24 ppm) compared to the LFG (0.41 ± 0.25 ppm; p > 0.05). The urine-to-serum fluoride ratio was significantly higher in the HFG (4.42 ± 3.47) than in the LFG (2.36 ± 1.40; p < 0.001). Non-anemic individuals had significantly higher hemoglobin levels (β = 2.51 g/dL, 95% CI: 1.96-3.07; p < 0.001).

Conclusion

Our findings suggest that the pattern of fluoride distribution and excretion, rather than total systemic fluoride concentration, may play a more critical role in influencing hemoglobin levels. The urine-to-serum fluoride ratio may serve as a reliable biomarker for estimating systemic fluoride burden and may be useful in monitoring fluoride exposure in vulnerable populations.

## Introduction

Anemia affects approximately one-third of the global population and is associated with increased morbidity and mortality, particularly among women and children [1]. It contributes to adverse birth outcomes, reduced work productivity in adults, and impaired cognitive and behavioral development in children [2]. According to the State Nutrition Profile, Telangana (September 2021), 12.65% of pregnant women in Telangana are reported to be anemic [3].

The World Health Organization (WHO) recommends a guideline value of 1.5 mg/L for fluoride concentration in drinking water, balancing its preventive benefits against potential toxicity. However, this is higher than the optimal range recommended for artificial fluoridation for dental caries prevention, which lies between 0.5 and 1.0 mg/L. In India, more conservative standards have been established: the Bureau of Indian Standards (BIS) prescribes an acceptable range of 0.6-1.2 mg/L, while both the Indian Council of Medical Research (ICMR) and the Committee on Public Health Engineering Manual and Code of Practice, Government of India, recommend an optimal concentration of 1.0 mg/L for drinking water [4,5]. 

Fluoride is widely distributed in the Earth's crust, mainly as fluorspar, fluorite, cryolite, and mica. Environmental sources include volcanic emissions, mineral weathering and groundwater dissolution, and industrial activities such as the manufacture of phosphate fertilizers and hydrofluoric acid. Dietary sources include fish, tea (especially brick tea), and fluoridated toothpaste. The fluoride content of drinking water varies regionally, with groundwater contamination being a major contributor to excessive fluoride intake. Elevated fluoride consumption can result in increased fluoride concentrations in both serum and urine. While urinary fluoride is commonly used as a biomarker of recent fluoride exposure, serum fluoride reflects the systemic fluoride burden [6]. 

In India, the most common cause of anemia is nutritional deficiency, including iron, vitamin B12, and folate deficiencies. However, fluoride exposure may also contribute to anemia. Fluoride has been shown to reduce vitamin B12 production and to impair intestinal nutrient absorption by damaging microvilli, leading to iron deficiency anemia. In such cases, even iron and folic acid supplementation may fail to correct anemia. Susheela et al. reported that increased urine fluoride levels were associated with decreased hemoglobin and that eliminating dietary fluoride led to improved hemoglobin levels, thereby helping to prevent anemia during pregnancy and adolescence [7,8]. In contrast, Eren E et al. observed no effect of chronic fluoride exposure on hemoglobin levels in experimental rat models [9]. Nonetheless, excess fluoride intake during pregnancy has been linked to increased prevalence and severity of anemia, and to adverse fetal outcomes including abortions, intra-uterine deaths, miscarriages, and congenital malformations [10].

Given this context, the present study was undertaken to evaluate the association between serum and urine fluoride levels and hemoglobin levels in pregnant women, after ruling out other causes of anemia.

## Materials and methods

Based on a study by Susheela et al (2016), assuming a pooled standard deviation of 0.9 units, the study required a sample size of 13 for each group (i.e. a total sample size of 26, assuming equal group sizes) to achieve a power of 80% and a level of significance of 5% (two sided) for detecting a true difference in means between the test and the reference group of -1 (i.e. 11 - 12) units [7]. 

A cross-sectional observational study was conducted from February 2023 to January 2024 involving pregnant women aged 18-45 years who had been residing for more than two years in villages with >4 ppm fluoride (high-fluoride group, HFG) or ≤1.5 ppm fluoride (low-fluoride group, LFG) and who were registered at the Department of Obstetrics at the All India Institute of Medical Sciences (AIIMS) Bibinagar during the first trimester of pregnancy (before 12 weeks). Subjects not from the study area, or with mental illnesses, addiction to alcohol or illegal drugs, smoking, or major medical or surgical history that could impact maternal and fetal outcomes were excluded from the study. All the pregnant women in the villages were contacted by ASHA (Accredited Social Health Activist) workers and were examined for antenatal health checkups and screening for anaemia.

**Figure 1:**
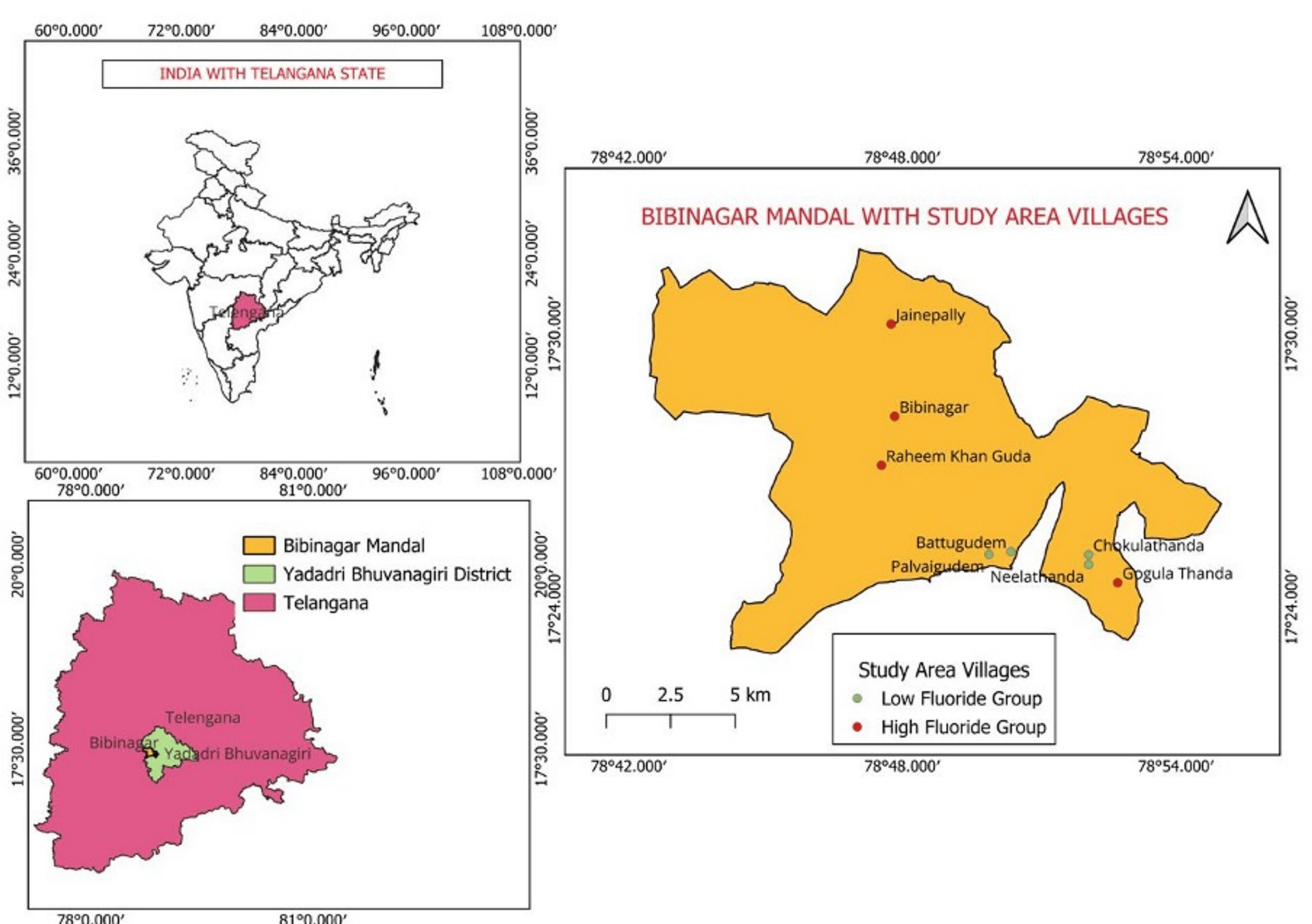
Study area map The study area map was generated by the authors using the QGIS Desktop software version 3.34.15 (https://qgis.org/).

The study population from the villages was classified into two groups based on the water fluoride levels reported on the government's website and located around the AIIMS, Bibinagar [11]. Group 1 - high-fluoride group (four villages with >4 ppm fluoride): 4 ppm in Bibinagar, 4.14 ppm in Gogula Thanda, 4.38 ppm in Raheemkhanguda, and 3.32 ppm in Jainepally. Group 2 - low-fluoride group (four villages with ≤1.5 ppm fluoride): Battugudem, Palvaigudem, Chokula Thanda, and Neela Thanda, with 1.6 ppm each.

Groundwater fluoride levels of the selected villages were considered. A detailed history of the study population was obtained, including age, gestational age, antenatal visits, previous pregnancies, dietary intake, nutritional supplements, and relevant medical and surgical history. Subjects with known cardiac, hepatic, renal, or pulmonary disorders; a history of acute or chronic infections, diabetes mellitus, malignancy; use of drugs affecting hematological parameters; or hereditary disorders were excluded. 

Written informed consent was obtained from each participant, and blood samples were collected (5 ml in EDTA tubes and 5 ml in serum-separator tubes) according to the guidelines provided by the Clinical and Laboratory Standards Institute (CLSI) [12]. A three-part automated hematology analyzer ( HORIBA ABX SAS, Yumizen H550, Montpellier, France) was used to measure complete blood counts and hemoglobin levels. The Complete Blood Picture (CBP) was reviewed to rule out other causes of anemia; for example, microcytic hypochromic anemia indicative of iron deficiency, and megaloblastic anemia seen in vitamin B12 and folic acid deficiencies. Additional haematological parameters, such as serum ferritin and total iron binding capacity (TIBC), and renal parameters were also recorded from anemic patients during their routine antenatal visits. One of the authors is a pathologist and screened all study participants for anaemia profile - hemoglobin (Hb), mean corpuscular volume (MCV), mean corpuscular hemoglobin (MCH), mean corpuscular hemoglobin concentration (MCHC), and red cell distribution width (RDW) and peripheral blood smear, as per WHO criteria for anaemia.

After 30 minutes of collection, the blood samples were centrifuged at 3000 rpm for 10 minutes using a refrigerated centrifuge, and the resulting supernatant (serum) was used to measure serum fluoride levels using an ion-selective electrode (Thermo Fischer Scientific Orion Star A214, Waltham, USA). For the preparation of reagents, only deionised water was used and not distilled water. For calibration, the concentrations of the standard solutions, namely, 0.1, 1.0, 10.0, and 100.0 ppm, were used. The sample and total ionic strength adjustment buffer (TISAB) III were mixed at a 1:20 dilution for serum and at a 1:10 dilution for urine and water samples for fluoride estimation. Calibration was considered acceptable when the average slope ranged from 54 to 60 mV.

Data were analyzed on SPSS software version 25 for Windows (IBM Corp, Armonk, USA). Continuous variables were expressed as mean ± standard deviation (SD) and compared using Student’s t test or Mann-Whitney U test, as appropriate. Categorical variables were presented as percentages and compared using the Chi-square test. Correlation analyses were performed using the Karl Pearson correlation coefficient on Jamovi version 2.6.24.0 (Jamovi Project, Sydney, Australia). Statistical significance was defined as p < 0.05, and the power of the test used for sample size calculation was 80%.

The Institutional Ethics Committee of All India Institute of Medical Sciences, Bibinagar, approved the study (no. AlIMS/BBN/IEC/JULY/2022/189).

## Results

A total of 98 pregnant women were included in the study, with 49 participants each in the HFG and LFG based on their village of residence. The majority of the participants were housewives (85.7%), while five were agricultural workers, of whom three belonged to HFG and two to LFG. None of the participants reported alcohol consumption or use of smoked tobacco products. Only one participant reported chewing betel nuts regularly. 

The HFG exhibited a slightly lower mean hemoglobin level compared to the LFG (11.80 ± 1.46g/dl in HFG vs 11.98 ± 1.46 g/dl in LFG) and a higher prevalence of anemia (24.48% vs 14.28%, respectively), although these differences were not statistically significant. There was no significant difference between the groups in terms of RBC count, packed cell volume (PCV), total leukocyte count (TLC), and other red blood cell indices. However, the platelet count was found to be significantly higher in the HFG (2.82 ± 0.61 x 10^3^/µL) compared to the LFG (2.58 ± 0.52 x 10^3^/µL; p=0.04) (Table 1).

**Table 1:** Baseline characteristics, blood indices and serum and urine fluoride levels of the study groups. P-value < 0.05 was considered statistically significant; ^*^z test was applied; ^†^t test was applied; Mann-Whitney U test values are not displayed in SPSS; only p-values are provided. Bold figures indicate p-value <0.05. SBP: Systolic blood pressure; DBP: Diastolic blood pressure; RBC: Red blood cell; PCV: Packed cell volume; MCV: Mean corpuscular volume; MCH: Mean corpuscular hemoglobin; MCHC: Mean corpuscular hemoglobin concentration; RDW: Red cell distribution width; TLC: Total leukocyte count.

Characteristic	Total sample size (n=98) (Mean±SD)	High-fluoride group (n=49) (Mean±SD)	Low-fluoride group (n=49) (Mean±SD)	t test/Mann-Whitney U test	p-value (Mann-Whitney U test)
Age (years)	23.78 ± 3.12	24.53 ± 3.12	23.02 ± 2.97	-	0.009
SBP (mm Hg)	106.56 ± 10.33	108.57 ± 11.50	104.55 ± 8.67	-	0.062
DBP (mm Hg)	70.13 ± 3.15	70.90 ± 6.49	69.37 ± 5.74	-	0.112
Hemoglobin (g/dl)	11.89 ± 1.45	11.80 ± 1.46	11.98 ± 1.46	-	0.363
Prevalence of Anemia (%)	19.38	24.48	14.28	1.632*	0.201*
RBC count (millions/mm^3^)	4.41 ± 0.52	4.41 ± 0.49	4.41 ± 0.57	-0.008^†^	0.994^†^
PCV (%)	35.10 ± 4.06	34.90 ± 3.98	35.31 ± 4.16	-0.501^†^	0.617^†^
MCV (fl)	79.68 ± 7.69	79.16 ± 7.62	80.21 ± 7.81	-	0.222
MCH (pg)	27.36 ± 3.51	26.98 ± 3.31	27.75 ± 3.69	-	0.133
MCHC (g/dl)	33.99 ± 1.58	33.88 ± 1.56	34.09 ± 1.62	-	0.193
RDW (%)	14.63 ±2.27	14.50 ± 1.84	14.77 ± 2.64	-	0.952
TLC (cells/µL)	7946.94 ± 2059.35	8327.76 ± 2449.59	7566.12 ± 1507.86	1.853^†^	0.068^†^
Granulocytes (%)	68.28 ± 9.66	68.45 ± 9.09	68.12 ± 10.29	-	0.782
Lymphocytes (%)	25.90 ± 9.68	25.55 ± 8.99	26.25 ± 10.41	-	0.828
Monocytes (%)	5.89 ± 2.04	5.88 ± 1.94	5.908 ± 2.16	-	0.771
Platelet count (10³/µL)	2.705 ± 0.58	2.82 ± 0.61	2.58 ± 0.52	-	0.040
Serum fluoride (ppm)	0.42 ± 0.24	0.37 ± 0.24	0.41 ± 0.25	-1.876^†^	0.064^†^
Urine fluoride (ppm)	1.19 ± 0.85	1.28 ± 0.81	1.09 ± 0.89	-	0.287
Urine-to-serum fluoride ratio	3.39± 2.83	4.42 ± 3.47	2.36± 1.40	-	0.001

Urine fluoride levels were higher in the HFG (1.28 ± 0.81 ppm) than in the LFG (1.09 ± 0.89 ppm), though this difference was not statistically significant (p>0.05). Interestingly, the serum fluoride levels were slightly lower in the HFG (0.37 ± 0.24ppm) compared to the LFG (0.41 ± 0.25 ppm), also without statistical significance (p>0.05). The urine fluoride to serum fluoride ratio was significantly higher in the HFG (4.42 ± 3.47) compared to the LFG (2.36 ± 1.40; p<0.001), indicating increased renal excretion of fluoride in chronically exposed individuals (Table 1). 

Correlation analysis revealed weak and statistically insignificant relationships between hemoglobin levels and fluoride levels in both urine and serum, suggesting no direct association between fluoride exposure and anemia. In contrast, a strong positive correlation was observed between serum and urine fluoride levels, and a moderate positive correlation was noted between urine fluoride and the urine-to-serum fluoride ratio, highlighting the role of the kidneys in systemic fluoride handling. However, a moderate negative correlation was observed between serum fluoride levels and the urine-to-serum fluoride ratio, suggesting inverse dynamics between circulating and excreted serum fluoride levels (Table2).

**Table 2:** Correlation matrix: correlation between hemoglobin levels and fluoride levels *p < 0.05; **p < 0.01; ***p < 0.001 df: degree of freedom

		Hemoglobin	Serum fluoride	Urine fluoride	Urine-to-serum fluoride ratio
Hemoglobin	Pearson's r	—			
	df	—			
	p-value	—			
Serum fluoride	Pearson's r	0.013	—		
	df	96	—		
	p-value	0.902	—		
Urine fluoride	Pearson's r	-0.021	0.601***	—	
	df	96	96	—	
	p-value	0.836	<0.001	—	
Urine-to-serum fluoride ratio	Pearson's r	0.037	-0.342***	0.360***	—
	df	96	96	96	—
	p-value	0.717	<0.001	<0.001	—

Multivariable linear regression analysis was performed to evaluate factors associated with hemoglobin concentration (N = 98). The overall model was statistically significant (F(4,93) = 23.4, p < 0.001) and explained 50.2% of the variance in hemoglobin (R² = 0.502; adjusted R² = 0.480). Hemoglobin category emerged as the strongest independent predictor, with individuals in the non-anaemic category demonstrating significantly higher hemoglobin levels compared to the anemic category (β = 2.51 g/dL, 95% CI: 1.96-3.07, p < 0.001). Age was not independently associated with hemoglobin (β = 0.039 g/dL per year, p = 0.274). Neither urinary fluoride category (β = −0.12, p = 0.572) nor fluoride exposure group (high versus normal fluoride; β = −0.03, p = 0.901) showed significant independent associations with hemoglobin after adjustment. Collinearity diagnostics indicated no multicollinearity (VIF range: 1.03-1.10), and influence statistics did not suggest undue impact of individual observations (Table 3).

**Table 3:** Multivariable linear regression of haemoglobin concentration adjusted for age, fluoride category, and exposure group Exposure group: 1 = high-fluoride group, 2 = low-fluoride group. Urine fluoride category: 0 = <1 ppm, 1 = >1 ppm. Hemoglobin category: 1 = anemic, 2 = non-anemic. ^*^Represents reference level.

			95% Confidence Interval				
Predictor	Estimate	Standard Error	Lower	Upper	t	p	Standard Estimate
Intercept^*^	8.9965	0.8858	7.2375	10.756	10.156	< 0.001>	
Age	0.0394	0.0358	-0.0317	0.111	1.100	0.274	0.0845
Exposure Group:							
2 – 1	-0.0279	0.2229	-0.4706	0.415	-0.125	0.901	-0.0191
Urine Fluoride Category:							
1 – 0	-0.1231	0.2169	-0.5538	0.308	-0.568	0.572	-0.0844
Hemoglobin Category:							
2 – 1	2.5141	0.2779	1.9622	3.066	9.047	<0.001	1.7241

Principal component analysis (PCA) was performed on serum fluoride, urine fluoride, and the urine-to-serum fluoride ratio to identify underlying dimensions of fluoride exposure. Using varimax rotation, two components with eigenvalues greater than 1 were retained, together explaining 96.0% of the total variance. The first component (PC1) accounted for 53.4% of the variance and was characterized by strong positive loadings of serum fluoride (0.901) and urine fluoride (0.889), representing systemic fluoride load. The second component (PC2) explained 42.7% of the variance and was dominated by the urine-to-serum fluoride ratio (0.985), reflecting a distinct fluoride handling or excretion pattern independent of total fluoride burden. The orthogonal structure of the components indicates that fluoride load and fluoride handling represent separate latent dimensions within the dataset (Table 4).

**Table 4:** Principal component analysis of fluoride exposure variables Component 1: Fluoride load (serum and urine fluoride). Component 2: Fluoride handling (urine-to-serum fluoride ratio). ^*^Varimax rotation was used.

Component Loadings^*^			
	Component		
	1	2	Uniqueness
Serum Fluoride	0.901	-0.379	0.0449
Urine Fluoride	0.889	0.406	0.0458
Urine fluoride/Serum fluoride Ratio		0.985	0.0288

Summary			
Component	SS Loadings	% of Variance	Cumulative %
1	1.60	53.4	53.4
2	1.28	42.7	96.0

Among the study participants, 32.7% were primigravida overall (26.5% in the HFG and 38.8% in the LFG). A history of previous abortions was reported by 24.5% in the HFG compared to 10.2% in the LFG (p=0.062). Additionally, a history of one intrauterine death, one still birth, and one premature birth was reported in the high-fluoride village group, with none reported in the low-fluoride village group.

A total of 84 participants (85.7%) reported using mineral water for drinking and cooking purposes, of whom 46 participants (93.9%) belonged to the LFG. A majority of participants (63.3%) purchased at least one can of water for daily cooking or drinking needs. Additionally, 76.5% of participants reported using filtered water. Among them, 36.7% of participants from HFG used candle filters, which was significantly higher than the 8.2% in the LFG (p<0.001). In contrast, 69.4% of LFG participants reported boiling water, compared to 18.4% in the HFG (p <0.001). Both groups reported purchasing vegetables from local markets, with 90.8% of total participants indicating that the vegetables were locally grown.

**Figure 2:**
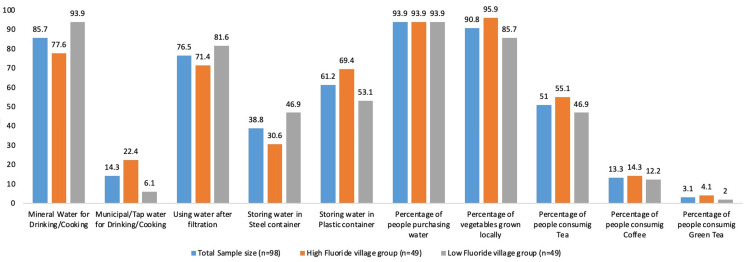
Sources of fluoride among study groups Y-axis: percentage

Regarding oral hygiene practices, 59.2% of HFG participants used Colgate Herbal toothpaste (Colgate-Palmolive, Mumbai, India) compared to 34.7% in the LFG (p <0.001). Overall, 46.9% of participants reported using Colgate Herbal toothpaste, followed by Dabur (17.3%) (Dabur, Ghaziabad, India), Colgate Tooth Powder (16.3%) (Colgate-Palmolive, Mumbai, India), and Close-Up (10.2%) (Hindustan Unilever, Mumbai, India). Only one participant reported using mouthwash (Listerine, JNTL Consumer Health (India), Powai, India), and only another participant reported the use of lipstick, mascara, foundation, or other makeup products. There was no statistically significant difference between the two groups in the daily consumption of beverages such as tea, coffee, and green tea (Figure 2).

## Discussion

Anemia in pregnancy is a multifactorial condition influenced by nutritional deficiencies, infections, genetic predisposition, and environmental exposures. Fluoride toxicity has been hypothesized to contribute to hematological alterations through impaired iron absorption, disruption of erythropoiesis, and oxidative stress-mediated red cell injury [13]. Experimental data suggest that high fluoride concentrations may interfere with iron metabolism and hemoglobin synthesis [14]. Observational studies from fluorosis-endemic regions have reported lower hemoglobin levels among chronically exposed individuals, particularly in vulnerable populations [6].

In the present study, pregnant women residing in high-fluoride villages exhibited a marginally lower mean hemoglobin concentration and a higher prevalence of anemia compared with those from low-fluoride villages; however, these differences were not statistically significant. Serum and urine fluoride concentrations were also comparable between the groups. Interestingly, the urine-to-serum fluoride ratio was significantly higher in the high-fluoride group, suggesting altered fluoride handling rather than increased systemic accumulation. This may reflect adaptive renal excretory mechanisms in chronically exposed individuals.

Correlation analysis revealed no significant association between hemoglobin concentration and either serum or urine fluoride levels. The strong positive correlation between serum and urine fluoride was physiologically expected, reflecting renal elimination of circulating fluoride. In contrast, the moderate inverse correlation between serum fluoride and the urine-to-serum ratio suggests dynamic regulation between circulating fluoride burden and excretory efficiency. These findings support the notion that fluoride exposure is not a unidimensional construct but involves both burden and handling components.

Multivariable linear regression analysis further clarified these relationships. After adjusting for age, exposure group, and urinary fluoride category, fluoride-related variables were not independently associated with hemoglobin concentration. Hemoglobin category emerged as the strongest predictor, consistent with its definitional relationship to measured hemoglobin values. Importantly, residence in a high-fluoride village did not independently predict hemoglobin concentration. Model diagnostics demonstrated low variance inflation factors and absence of influential outliers, confirming robustness of the analysis. These findings differ from those reported by Susheela et al., who described an inverse association between urinary fluoride and hemoglobin levels in fluorosis-affected populations [7]. Similarly, Goyal et al. observed increased anemia severity and adverse fetal outcomes with excess fluoride intake during pregnancy [10]. Conversely, Eren et al. reported no significant impact of chronic fluorosis on hemoglobin levels in experimental settings [9], findings more aligned with the present study.

To better characterise fluoride exposure patterns, principal component analysis was performed exclusively on fluoride biomarkers. Two orthogonal components were identified: systemic fluoride load, defined by serum and urine fluoride, and fluoride handling pattern, defined by the urine-to-serum fluoride ratio. These components together explained 96% of the variance, confirming that total fluoride burden and excretion dynamics represent distinct biological dimensions. The absence of hemoglobin levels within these components reinforces the regression findings that hemoglobin variability in this study population was not structurally linked to fluoride exposure constructs.

It is plausible that fluoride-related hematological effects are context-dependent and may become evident primarily in individuals with underlying nutritional deficiencies or hematological vulnerability. Pornprasert et al. reported that excessive fluoride intake may exacerbate anemia risk in individuals with iron deficiency, thalassemia, or glucose-6-phosphate dehydrogenase (G-6-PD) deficiency [15]. Participants with known causes of anemia were excluded from the present study, limiting extrapolation to these high-risk subgroups.

The cross-sectional design limits causal inference. Although the sample size was adequate for multivariable modeling, small effect sizes cannot be excluded. Dietary iron intake, inflammatory markers, and comprehensive micronutrient status were not assessed, which may confound subtle hematological effects. Additionally, fluoride exposure was assessed at a single time point and may not fully represent cumulative exposure dynamics.

## Conclusions

This study found no independent association between chronic environmental fluoride exposure and hemoglobin concentration in pregnant women within the observed exposure range. Although women from high fluoride villages demonstrated altered fluoride excretion patterns, these did not translate into significant hematological differences after adjustment for confounders. The findings suggest that systemic fluoride burden alone is unlikely to be a primary determinant of anemia during pregnancy in this population. Hemoglobin variability appears to be influenced more strongly by established nutritional and physiological factors rather than environmental fluoride exposure.

In areas with fluoridated water supplies or fluoride-contaminated groundwater, continued surveillance of environmental fluoride levels and access to safe drinking water remain essential to prevent long-term skeletal and dental fluorosis. Screening using the urine-to-serum fluoride ratio and community-level interventions, including appropriate water filtration practices and public health education, should remain integral components of preventive strategies in fluoride-endemic settings.
